# Real-world effectiveness and safety of lasmiditan in patients with migraine: an observational study

**DOI:** 10.3389/fneur.2026.1968634

**Published:** 2026-09-29

**Authors:** Saro Kobayashi, Shiho Suzuki, Mukuto Shioda, Tomohiko Shiina, Hiroaki Fujita, Hirotaka Sakuramoto, Ryotaro Hida, Keisuke Suzuki

**Affiliations:** 1 Department of Neurology, Dokkyo Medical University, Mibu, Japan; 2 Stroke Center, Dokkyo Medical University Hospital, Mibu, Japan

**Keywords:** adverse effects, lasmiditan, migraine, real-world study, treatment response

## Abstract

**Objective:**

To evaluate the real-world effectiveness and safety of lasmiditan and determine the factors associated with its effectiveness and adverse effects.

**Methods:**

We evaluated 75 migraine patients (15 males and 60 females; age 44.0 ± 12.4 years), including 20 (35.1%) with chronic migraine, who completed a questionnaire in a single-center setting. Headache severity before and after taking lasmiditan was scored using a numerical rating scale (NRS), and changes in pain and most bothersome symptoms (MBS) were assessed over time. Adverse effects such as drowsiness, weakness, and dizziness, as well as preferences, were also evaluated.

**Results:**

The response rate was 76% (57/75 patients, 180 attacks). Headache improvement was observed in 61.3% (100/144 attacks) at 2 h and 73.0% (119/151 attacks) at 24 h, with headache resolution rates of 27.8% (50/144 attacks) and 43.9% (71/151 attacks), respectively. Compared with patients who did not receive anti-CGRP monoclonal antibodies, patients who received anti-CGRP monoclonal antibodies had higher 2-h headache improvement rates (77.0% vs. 57.9%, *p* = 0.015). MBS improved by 56.9% at 60 min and 70.8% at 24 h. Adverse effects occurred in 81.3% of the patients, most commonly somnolence (39.4%), followed by weakness (32.2%) and dizziness (19.4%). Patients without somnolence had higher 24-h headache improvement rates than those with somnolence did (78.3% vs. 52.9%, *p* < 0.001). Despite frequent adverse effects, 25% of patients (19/75) used lasmiditan as their sole treatment for acute attacks.

**Conclusion:**

Lasmiditan demonstrated meaningful effectiveness in a real-world setting, although adverse effects were common.

## Highlights

- The rate of headache resolution 2 h after the use of lasmiditan was 27.8%.
- Headache improvement increased over time after lasmiditan administration.
- The absence of somnolence or weakness was associated with better headache outcomes.

## Introduction

According to the Global Burden of Disease Study of 2016, migraine is a common and disabling neurological disorder and is the second leading cause of disease burden worldwide (1). Migraine predominantly affects working-age individuals, particularly women, and significantly affects quality of life. Acute treatments include acetaminophen and nonsteroidal anti-inflammatory drugs (NSAIDs), as well as triptans, which are 5-HT_1B/1D_ receptor agonists (2). Triptans exert their therapeutic effects by stimulating 5-HT_1B/1D_ receptors, thereby suppressing the release of calcitonin gene-related peptide (CGRP). However, approximately 30–40% of patients do not respond to triptans (3), and the use of these drugs is limited by adverse effects and contraindications in patients with vascular diseases because their vasoconstrictive properties are mediated by 5-HT_1B_ receptor activation.

Lasmiditan is a selective 5-HT_1F_ receptor agonist and a novel acute migraine treatment that acts on the trigeminovascular system and suppresses CGRP release without vasoconstrictive effects (4). Because lasmiditan has greater central nervous system penetration than triptans do, it may be effective in triptan nonresponders because it acts on both the central and peripheral pathways (4). Although lasmiditan differs from conventional acute migraine medications and is being increasingly used in clinical practice, real-world data on its effectiveness and safety remain limited. In recent years, CGRP monoclonal antibodies (CGRP mAbs), which target CGRP or its receptors, have been increasingly used as preventive therapy. A previous study suggested that migraine patients who regularly use triptans have a high response rate to CGRP mAbs (5). As triptans and CGRP mAbs act on different parts of the CGRP pathway, their combined use may produce additive or synergistic effects. Similarly, lasmiditan is expected to show enhanced effectiveness when used in combination with CGRP mAbs; however, clinical evidence remains scarce (6). Therefore, in this study, we aimed to evaluate the real-world effectiveness and safety of lasmiditan.

## Methods

### Study design and setting

A retrospective single-center observational study using clinical and questionnaire data collected during routine clinical practice was conducted among migraine patients who were prescribed Lasmiditan at the neurology outpatient clinic at Dokkyo Medical University Hospital. This study was approved by the Ethics Committee of Dokkyo Medical University Hospital. In accordance with the Declaration of Helsinki, all participating patients were informed about this observational study and given the opportunity to opt out; therefore, individual written consent was waived.

### Diagnosis of migraine

Migraine without aura and migraine with aura were diagnosed on the basis of the criteria of the International Classification of Headache Disorders, 3rd Edition (ICHD-3) (7). In accordance with the ICHD-3 criteria, chronic migraine was defined as headache occurring ≥ 15 days per month for ≥ 3 months, with migraine features present ≥ 8 days per month. Medication overuse headache (MOH) was defined as headache occurring ≥ 15 days per month associated with regular use of the causative analgesic for ≥ 3 months.

### Patients

The study included migraine patients aged ≥18 years who received at least one prescription for lasmiditan at the neurology outpatient clinic of Dokkyo Medical University Hospital between September 2024 and December 2025. Of the 75 patients who received the questionnaire, 57 returned completed questionnaires and were included in the analysis (Figure 1). Among the 18 patients who were not included, five did not take lasmiditan after prescription, ten took lasmiditan but did not return the questionnaire, and three discontinued follow-up after prescription.

**Figure 1:**
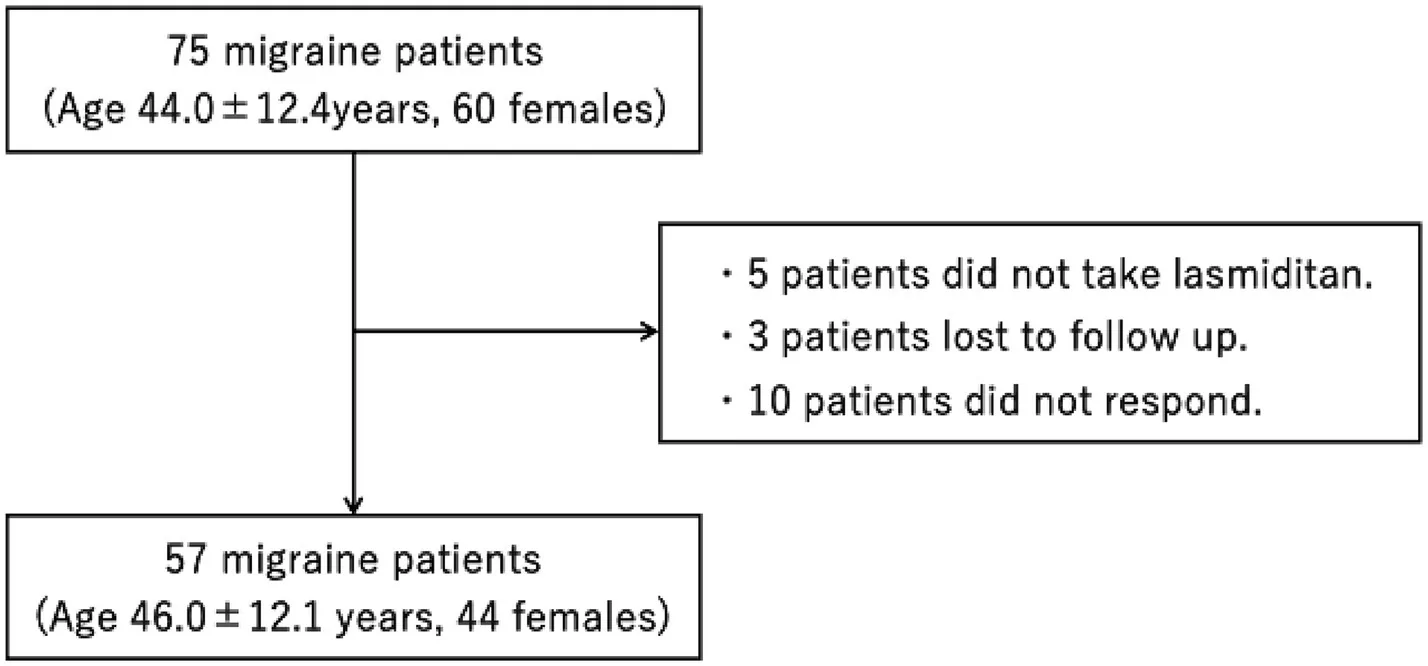
Flowchart of the study.

### Clinical evaluation and data extraction

A self-administered questionnaire was distributed to migraine patients at the time of their lasmiditan prescription, and patients completed one questionnaire per dose (a maximum of five per patient). Pain was evaluated using an 11-point numerical rating scale (NRS, 0–10) at baseline and at 30, 60, and 120 min and 24 h after administration. Most bothersome symptoms (MBS; e.g., photophobia, phonophobia, and nausea) were recorded as present or absent at the same time points. Adverse effects, including dizziness, somnolence, fatigue, lightheadedness, sensory disturbances, and palpitations, were recorded, along with free-text responses. Patient preferences regarding effectiveness and adverse effects were assessed using a 10-point scale. Clinical data, including migraine aura, accompanying symptoms, comorbidities, disease duration, and concomitant acute and preventive medications, were obtained from medical records.

### Definition of headache outcomes

Pain severity was defined as an NRS score of 0 (no headache), 1–3 (mild), 4–7 (moderate), or 8–10 (severe). Headache resolution was defined as a reduction from any pain level to an NRS score of 0. Headache improvement was defined as a reduction from moderate or severe pain to mild or no pain. Headache resolution and headache improvement rates were evaluated at 30, 60, and 120 min and 24 h after administration.

### Statistical analysis

Considering the real-world setting, formal sample size calculations were not performed. No imputation was performed for missing data. Analyses at each assessment time point were conducted using the available data, and the denominator therefore varied according to the number of available responses. The chi-square test or Fisher’s exact test was used for comparisons of categorical variables, as appropriate. A two-tailed *p* value of < 0.05 was considered to indicate statistical significance. IBM SPSS Statistics ver. 30 (IBM SPSS, Tokyo, Japan) was used for statistical analysis, and figures were created using Microsoft Excel version 16.108.

## Results

### Baseline characteristics

The clinical backgrounds of the patients are summarized in Table 1. A total of 180 headache episodes were reported. Nearly 80% of the respondents were female (44 females, 13 males), and the median duration of illness was 22 years. Twenty patients (35.1%) had chronic migraine, nine (15.8%) had aura, and twenty (35.1%) had MOH. More than 70% of the respondents reported some form of sensory hypersensitivity (photophobia, 78.9%; phonophobia, 77.2%; osmophobia, 70.2%). In addition to the use of lasmiditan, the most common acute treatment medications used in combination were triptans (73.7%) and NSAIDs (54.4%). Preventive treatments included anti-CGRP mAbs (50.9%), antidepressants (29.8%), and calcium channel blockers (26.3%). The most common dose of lasmiditan was 50 mg (68.4%). Baseline characteristics were generally comparable between the included and non-included patients, except for a higher prevalence of medication overuse headache (MOH) in the non-included group (66.7% vs. 35.1%, *p* = 0.018; Supplementary Table 4).

**Table 1:** Patient characteristics.

	Total
*n* (M/F)	57 (13/44)
Number of headaches	180
Age, years	46.0 ± 12.1
Disease duration, years	22.0 ± 11.5
Chronic migraine, *n* (%)	20 (35.1)
Migraine with aura, *n* (%)	9 (15.8)
MOH, n (%)	20 (35.1)
Accompanying symptoms, *n* (%)	
Photophobia	45 (78.9)
Phonophobia	44 (77.2)
Osmophobia	40 (70.2)
Analgesics, *n* (%)	
NSAIDs	31 (54.4)
Acetaminophen	6 (10.5)
Triptans	42 (73.7)
Migraine prophylaxis, *n* (%)	
CGRP mAbs	29 (50.9)
Fremanezumab	13 (22.8)
Galcanezumab	12 (21.1)
Erenumab	4 (7.0)
Antidepressants	17 (29.8)
Calcium channel blockers	15 (26.3)
Antiepileptics	11 (19.3)
Lasmiditan dose	
50 mg	39 (68.4)
100 mg	17 (29.8)
200 mg	1 (1.8)
Comorbidities	43 (75.4)

CGRP, calcitonin gene-related peptide; mAbs, monoclonal antibodies; MOH, medication overuse headache; NSAIDs, nonsteroidal anti-inflammatory drugs.

### Changes in pain after taking lasmiditan

The changes in headache before and after the use of lasmiditan are shown in Figure 2. The headache improvement rates were 48.5, 61.3 and 73.0% at 60 min, 120 min, and 24 h, respectively, while the resolution rates were 12.8, 27.8, and 43.9%, respectively. The changes in headache according to CGRP mAb use are shown in Figure 3. The headache improvement rate at 120 min was significantly greater in the CGRP mAb group than in the nonuse group (77.0% vs. 57.9%, *p* = 0.015), with no significant differences observed at other time points. In contrast, there were no significant differences in headache resolution rates between the groups at any time point (Table 2). We also compared outcomes according to adverse events. Patients without somnolence had a significantly greater headache improvement rate at 24 h than those with somnolence did (52.9% vs. 78.3%, *p* < 0.001), although headache resolution rates did not differ between the groups (Supplementary Table 1). Similarly, patients without weakness had significantly higher headache resolution rates at 2 and 24 h, whereas the headache improvement rates did not differ between the groups (Supplementary Table 2). No significant difference in headache improvement or resolution rates was observed according to the presence or absence of dizziness or lightheadedness.

**Figure 2:**
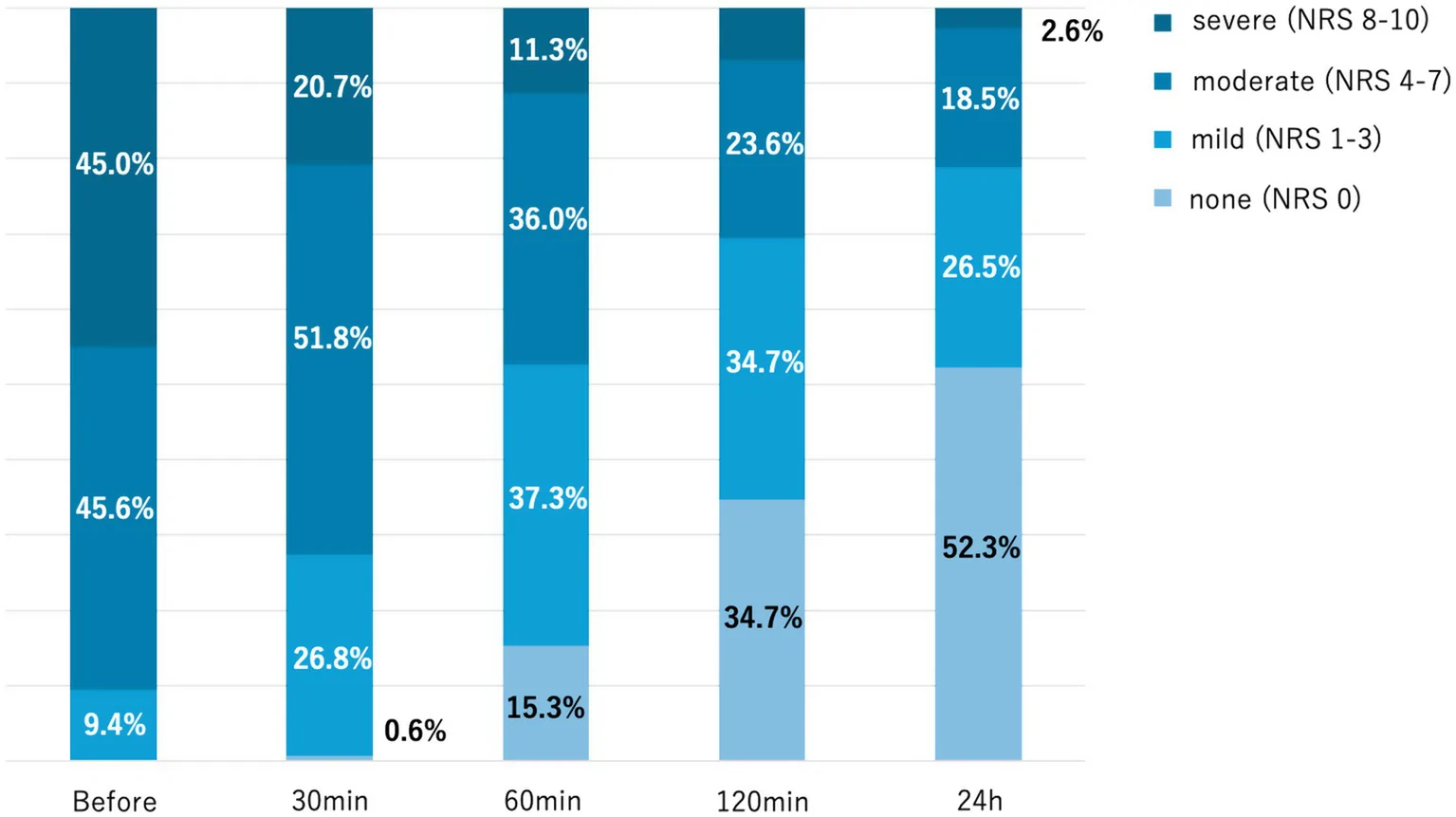
Changes in headache intensity after taking lasmiditan.

**Figure 3:**
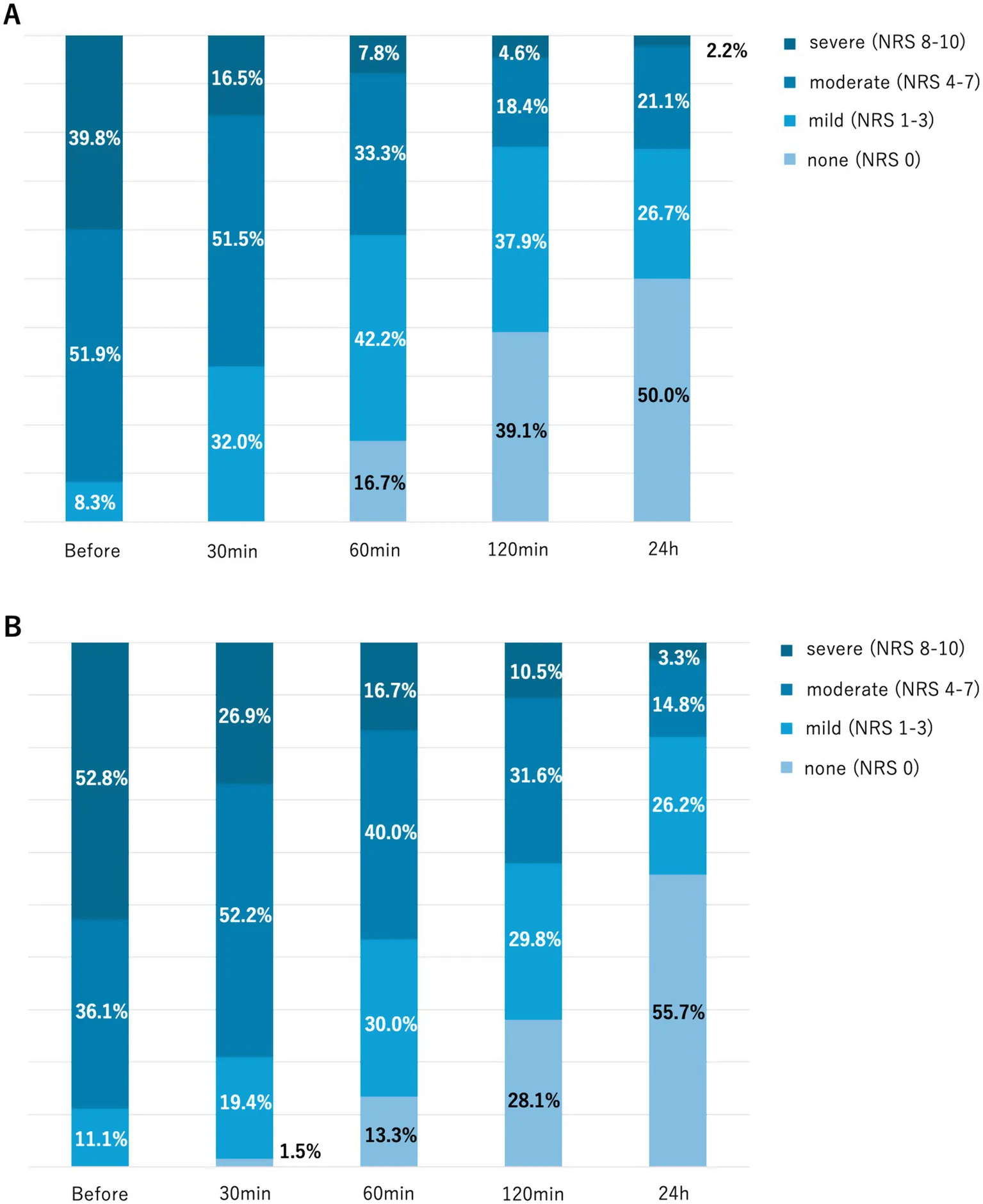
Comparison of changes in headache intensity after taking lasmiditan on the basis of the presence or absence of CGRP mAbs. Changes in headache intensity after lasmiditan use in patients who received CGRP mAbs **(A)** and those who did not receive CGRP mAbs **(B)**.

**Table 2:** Comparison of headache resolution rates and improvement rates based on the use of CGRP mAbs.

	30 min	60 min	120 min	24 h
Improvement rate				
CGRP mAbs +	(31/97) 32.0%	(53/90) 58.9%	(67/87) 77.0%	(69/90) 76.7%
CGRP mAbs −	(14/67) 20.9%	(26/60) 43.3%	(33/57) 57.9%	(50/61) 82.0%
*p* value	0.119	0.061	**0.015**	0.434
Resolution rate				
CGRP mAbs +	(0/97) 0.0%	(15/90) 16.7%	(34/87) 39.1%	(45/90) 50.0%
CGRP mAbs −	(1/67) 1.5%	(8/60) 13.3%	(16/57) 28.1%	(34/61) 55.7%
*p* value	0.409	0.579	0.175	0.489

Statistically significant values are shown in bold. CGRP, calcitonin gene-related peptide.

### Changes in MBS after lasmiditan administration

There were 72 attacks with MBS at baseline. MBS resolution was observed in 21 (29.2%), 41 (56.9%), 42 (58.3%), and 51 (70.8%) attacks at 30, 60, and 120 min and 24 h, respectively (Supplementary Table 3).

### Adverse events associated with lasmiditan

Among the 57 respondents, 43 (75.4%) reported at least one adverse effect, accounting for 105 of 180 headache episodes (58.3%). The reported adverse effects are shown in Table 3. The most frequent adverse effect was somnolence (39.4%), followed by weakness (32.2%), dizziness (19.4%), and lightheadedness (17.8%).

**Table 3:** Adverse events.

Symptoms	
Somnolence, *n* (%)	71 (39.4)
Weakness, *n* (%)	58 (32.2)
Dizziness, *n* (%)	35 (19.4)
Unsteadiness, *n* (%)	32 (17.8)
Paresthesia, *n* (%)	10 (5.6)
Palpitations, *n* (%)	5 (2.8)
Nausea, *n* (%)	4 (2.2)
Tinnitus, *n* (%)	2 (1.1)

Multiple answers were allowed.

### Drug preferences

Although adverse effects were common, the mean score for the item where patients selected their preference for the drug on a 10-point scale from 1 (dislike) to 10 (like) was 5.5 ± 2.3, indicating that a certain number of patients preferred the drug. Furthermore, one-quarter of patients reported preferring lasmiditan over acetaminophen, NSAIDs, or triptans. At the time of response collection, 25% of patients (19 of 75) were using lasmiditan alone as an acute treatment for migraines.

## Discussion

This study investigated the usefulness and side effects of lasmiditan in clinical practice using a dedicated questionnaire. The main findings of this study are as follows. (1) The headache resolution rate 2 h after taking lasmiditan was 27.8%. (2) Approximately 75% of those taking the drug reported at least one adverse effect, with somnolence (39.4%), weakness (32.2%), dizziness (19.4%), and lightheadedness (17.8%) being the most common. (3) Compared with patients who did not receive CGRP mAbs, patients who received CGRP mAbs as preventive therapy had a significantly greater headache improvement rate at 2 h after they received lasmiditan (77.0% vs. 57.9%, *p* = 0.015; Table 2). (4) Compared with patients who reported somnolence, patients who did not report somnolence as an adverse effect had a significantly higher headache improvement rate at 24 h after taking lasmiditan (Supplementary Table 1). Furthermore, compared with patients who reported weakness, patients who did not report weakness as an adverse effect had significantly higher headache resolution rates at both 2 and 24 h after administration (Supplementary Table 2). Although most baseline characteristics were comparable between the included and non-included patients, MOH was more frequent in the non-included group, and therefore attrition bias cannot be completely excluded.

The 2-h headache resolution rate after lasmiditan administration has varied across previous studies. In phase 3 trials, including SAMURAI, SPARTAN, CENTRION, and GLADIATOR, the 2-h headache resolution rates generally ranged from approximately 26 to 39%, with higher doses associated with better efficacy (8–11). In contrast, real-world studies from Japan and Korea reported higher 2-h headache resolution rates of approximately 45–48% (12, 13). In the present study, the prevalence of headache at 2 h was 27.8%, which may be partly explained by the high proportion of patients receiving the 50 mg dose and the inclusion of more refractory patients, including those with chronic migraine and MOH. Nevertheless, lasmiditan showed a certain level of effectiveness. Even in this severe cohort, the headache improvement and resolution rates were 61.3 and 27.8% at 2 h and 73.0 and 43.9% at 24 h after administration, respectively. Supplementary Table 1 shows the changes in headache intensity after taking lasmiditan in the CM and the MOH group. Comparisons between the CM and EM groups and between the MOH and non-MOH groups revealed that the EM group exhibited higher rates of headache improvement and resolution 24 h after administration, while the non-MOH group showed significantly higher rates of headache resolution at 2 h post-administration, as well as higher rates of headache improvement at both 2 and 24 h post-administration (Supplementary Table 5).

There is still no clear consensus regarding the predictors of the response to lasmiditan. Tanei et al. (14) reported that responders were more likely to be male, have chronic migraine, use calcium channel blockers, and receive CGRP mAb treatment. In univariate, but not multivariate, analyses, Jeong et al. (13) reported that triptan responsiveness predicted the lasmiditan response. Other real-world studies have not identified clear predictors (12, 15). Although this study did not examine the relationship between triptan and lasmiditan responsiveness, both drugs are 5-HT receptor agonists that act on the CGRP pathway, suggesting that responders may share common characteristics. In contrast, a network meta-analysis of triptan-insufficient responders revealed that oral lasmiditan (200 mg) showed the greatest analgesic efficacy among lasmiditan, rimegepant, and ubrogepant (16). Triptan insufficiency has been associated with central sensitization and genetic variability in the expression of serotonin receptors, such as 5-HT_1_ receptors, and neurotransmitter metabolism (17, 18). However, a subanalysis of the CENTURION trial revealed no association between responsiveness to lasmiditan and triptan (19). Previous studies have also shown high response rates to CGRP mAbs among triptan users (5). In the present study, concomitant CGRP mAbs use was associated with greater headache improvement following lasmiditan treatment. However, given the observational design and potential differences in patient characteristics, this finding should not be interpreted as evidence of an additive or synergistic effect.

The types and frequencies of side events in this study were generally consistent with those in previous reports. Although adverse events were common, a certain number of patients evaluated lasmiditan favorably, which is consistent with the findings reported by Ishii et al. (12). In the present study, many participants were limited to a single use because of adverse events. However, a previous study showed that administration after meals slows the increase in the plasma lasmiditan concentration and reduces both the frequency and severity of adverse events, suggesting that appropriate guidance regarding medication timing may improve tolerability and adherence (20). We compared baseline clinical characteristics between patients with and without somnolence and between those with and without weakness (Supplementary Table 6). Patients without somnolence had a significantly higher prevalence of medication-overuse headache (MOH) than those with somnolence, whereas no significant differences were observed in the other baseline characteristics. This suggests that MOH status may represent a potential confounding factor in the association between somnolence and treatment response. In contrast, no significant differences in baseline clinical characteristics were observed between patients with and without weakness. Furthermore, in both groups, regardless of whether adverse events occurred, there was no difference in the use of preventive medications, including anti-CGRP antibodies.

Lasmiditan is highly lipid soluble and readily penetrates the central nervous system (6). Although the precise mechanism underlying central nervous system-related adverse events remains unclear, these symptoms may be mediated by central 5-HT_1F_ receptors, as their onset closely parallels the increase in plasma lasmiditan concentration and appears to be dose dependent (21). Post hoc analyses of the SAMURAI, SPARTAN, MONONOFU, and CENTURION trials suggested that patients who experienced central nervous system-related adverse events, such as somnolence, weakness, and dizziness, tended to show better headache improvement 2 h after administration, although the differences were not statistically significant (22). Interestingly, in the present study, patients without somnolence or weakness showed higher rates of headache improvement and headache freedom at both 2 and 24 h after administration. These findings suggest that the therapeutic effects of lasmiditan and its central nervous system-related adverse events may not necessarily involve the same mechanisms. Because lasmiditan readily penetrates the central nervous system, both its therapeutic effects and adverse events are thought to involve central 5-HT_1F_ receptor activity (23). However, the present findings suggest that these effects may not necessarily occur in parallel. Further studies are needed to clarify the relationship between the efficacy and adverse events associated with lasmiditan. Somnolence, weakness, and dizziness may adversely affect alertness, mobility, and daily functioning and therefore require appropriate counseling and individualized dosing. Despite the relatively high frequency of these adverse events, lasmiditan may remain a useful acute treatment option, particularly in patients with an insufficient response to NSAIDs or triptans or in those for whom triptans are contraindicated.

This study has several limitations. First, it is a single-center study with a small number of participants, which may limit its statistical power. Given that this study was conducted in the unique environment of a university hospital headache clinic, the proportion of patients with chronic migraines or multiple comorbidities may have been greater than that typically seen in general clinical practice. Therefore, the study population may have included patients with more severe disease, and caution is warranted when interpreting the results. Furthermore, the study design does not distinguish between new and existing users of lasmiditan, and the decision to prescribe was left to the discretion of the attending physician, potentially leading to selection bias. Additionally, since participants could have responded between one and five times, patients who tolerated or preferred lasmiditan may have contributed disproportionately to the responses, potentially influencing the results. Moreover, 18 of the 75 patients who received the questionnaire were not included in the analysis, which may have introduced attrition bias. Finally, because this study was conducted at a single university hospital in Japan, differences in healthcare access, treatment availability, prescribing practices, reimbursement systems, and socioeconomic factors may limit the generalizability of our findings to other populations and healthcare systems.

## Conclusion

In this study, we evaluated the efficacy and side effects of lasmiditan in clinical practice. Although more than 70% of patients experienced adverse events, lasmiditan demonstrated clinically meaningful headache relief, and approximately one-quarter of patients rated it as more effective than acetaminophen, NSAIDs, or triptans.

## Data Availability

The raw data supporting the conclusions of this article will be made available by the authors, without undue reservation.

## References

[1] Disease GBD Injury I Prevalence C. Global, regional, and national incidence, prevalence, and years lived with disability for 328 diseases and injuries for 195 countries, 1990-2016: a systematic analysis for the global burden of Disease study 2016. Lancet. (2017) 390:1211–59. doi: 10.1016/S0140-6736(17)32154-2, 28919117 PMC5605509

[2] Puledda F Sacco S Diener HC Ashina M Al-Khazali HM Ashina S . International headache society global practice recommendations for the acute pharmacological treatment of migraine. Cephalalgia. (2024) 44:3331024241252666. doi: 10.1177/03331024241252666, 39133176

[3] Viana M Genazzani AA Terrazzino S Nappi G Goadsby PJ. Triptan nonresponders: do they exist and who are they? Cephalalgia. (2013) 33:891–6. doi: 10.1177/0333102413480756, 23564209

[4] Nelson DL Phebus LA Johnson KW Wainscott DB Cohen ML Calligaro DO . Preclinical pharmacological profile of the selective 5-HT1F receptor agonist lasmiditan. Cephalalgia. (2010) 30:1159–69. doi: 10.1177/0333102410370873, 20855361

[5] Vollono C Romozzi M Munafo A Vigani G De Cesaris F Calabresi P . Influence of triptans use on anti-CGRP mAbs response: a prospective, cohort study. J Neurol. (2025) 272:468. doi: 10.1007/s00415-025-13202-0, 40536712

[6] Clemow DB Johnson KW Hochstetler HM Ossipov MH Hake AM Blumenfeld AM. Lasmiditan mechanism of action—review of a selective 5-HT(1F) agonist. J Headache Pain. (2020) 21:71. doi: 10.1186/s10194-020-01132-3, 32522164 PMC7288483

[7] Headache Classification Committee of the International Headache Society (IHS). The international classification of headache disorders. Cephalalgia. (2018) 38:1–211. doi: 10.1177/0333102417738202 29368949

[8] Kuca B Silberstein SD Wietecha L Berg PH Dozier G Lipton RB . Lasmiditan is an effective acute treatment for migraine: a phase 3 randomized study. Neurology. (2018) 91:e2222–32. doi: 10.1212/WNL.0000000000006641, 30446595 PMC6329326

[9] Ashina M Reuter U Smith T Krikke-Workel J Klise SR Bragg S . Randomized, controlled trial of lasmiditan over four migraine attacks: findings from the CENTURION study. Cephalalgia. (2021) 41:294–304. doi: 10.1177/0333102421989232, 33541117 PMC7961651

[10] Brandes JL Klise S Krege JH Case M Khanna R Vasudeva R . Long-term safety and efficacy of lasmiditan for acute treatment of migraine: final results of the GLADIATOR study. Cephalalgia Rep. (2020) 3:2515816320958176. doi: 10.1177/2515816320958176 PMC6779019 31433669

[11] Goadsby PJ Wietecha LA Dennehy EB Kuca B Case MG Aurora SK . Phase 3 randomized, placebo-controlled, double-blind study of lasmiditan for acute treatment of migraine. Brain. (2019) 142:1894–904. doi: 10.1093/brain/awz134, 31132795 PMC6620826

[12] Ishii R Ishizuchi K Watanabe N Fukazawa R Trivedi M Nakahara J . Efficacy and tolerability of 100 mg of lasmiditan for migraine: a multi-center, prospective observational real-world study in Japan. Cephalalgia. (2024) 44:3331024241258695. doi: 10.1177/03331024241258695, 38859749

[13] Jeong HJ Lee MJ. Real-world experience of Lasmiditan for the acute treatment of migraine. J Clin Neurol. (2025) 21:332–9. doi: 10.3988/jcn.2025.0021, 40635538 PMC12303685

[14] Tanei T Yamamoto S Maesawa S Nishimura Y Ishizaki T Nagashima Y . Identifying factors associated with the efficacy of Lasmiditan 50 mg as an acute treatment for migraine attacks under various dosing conditions in real-world clinical practice. Neurol Int. (2025) 17:62. doi: 10.3390/neurolint17050062, 40423218 PMC12114562

[15] Shibata Y Sato H Sato A Harada Y. Efficacy of Lasmiditan as a secondary treatment for migraine attacks after unsuccessful treatment with a triptan. Neurol Int. (2024) 16:643–52. doi: 10.3390/neurolint16030048, 38921952 PMC11206899

[16] Laohapiboolrattana W Jansem P Anukoolwittaya P Roongpiboonsopit D Hiransuthikul A Pongpitakmetha T . Efficacy of lasmiditan, rimegepant and ubrogepant for acute treatment of migraine in triptan insufficient responders: systematic review and network meta-analysis. J Headache Pain. (2024) 25:194. doi: 10.1186/s10194-024-01904-1, 39516789 PMC11544997

[17] Christensen AF Esserlind AL Werge T Stefansson H Stefansson K Olesen J. The influence of genetic constitution on migraine drug responses. Cephalalgia. (2016) 36:624–39. doi: 10.1177/0333102415610874, 26502740

[18] Gentile G Borro M Lala N Missori S Simmaco M Martelletti P. Genetic polymorphisms related to efficacy and overuse of triptans in chronic migraine. J Headache Pain. (2010) 11:431–5. doi: 10.1007/s10194-010-0241-0, 20652353 PMC3452274

[19] Reuter U Krege JH Lombard L Gomez Valderas E Krikke-Workel J Dell-Agnello G . Lasmiditan efficacy in the acute treatment of migraine was independent of prior response to triptans: findings from the CENTURION study. Cephalalgia. (2022) 42:20–30. doi: 10.1177/03331024211048507, 34644189

[20] Tatsuoka Y Naruse T Shima A Takahashi R Tatsuoka Y. Comparison of the adverse events of preprandial and postprandial lasmiditan intake: a retrospective chart review. Cephalalgia Rep. (2025) 8:25158163251355591. doi: 10.1177/25158163251355591

[21] Tepper SJ Krege JH Lombard L Asafu-Adjei JK Dowsett SA Raskin J . Characterization of dizziness after lasmiditan usage: findings from the SAMURAI and SPARTAN acute migraine treatment randomized trials. Headache. (2019) 59:1052–62. doi: 10.1111/head.13544, 31152441

[22] Doty EG Hauck PM Krege JH Komori M Hake AM Dong Y . The association between the occurrence of common treatment-emergent adverse events and efficacy outcomes after Lasmiditan treatment of a single migraine attack: secondary analyses from four pooled randomized clinical trials. CNS Drugs. (2022) 36:771–83. doi: 10.1007/s40263-022-00928-y, 35779194 PMC9259541

[23] Labastida-Ramirez A Rubio-Beltran E Haanes KA Chan KY Garrelds IM Johnson KW . Lasmiditan inhibits calcitonin gene-related peptide release in the rodent trigeminovascular system. Pain. (2020) 161:1092–9. doi: 10.1097/j.pain.0000000000001801, 31977930 PMC7170441

