# Supplementary material for: Real-world effectiveness and safety of lasmiditan in patients with migraine: an observational study
Source: Front Neurol. 2026 Sep 29;17:1968634. doi: 10.3389/fneur.2026.1968634 (PMC13657970; doi:10.3389/fneur.2026.1968634)
Supplement: Supplementary file 1 [file Supplementary_file_1.docx]

**Supplementary Figure 1.** Changes in headache intensity after taking lasmiditan in the CM and the MOH group.

**CM group**

**MOH group**
