# Supplementary material for: Real-world effectiveness and safety of lasmiditan in patients with migraine: an observational study
Source: Front Neurol. 2026 Sep 29;17:1968634. doi: 10.3389/fneur.2026.1968634 (PMC13657970; doi:10.3389/fneur.2026.1968634)
Supplement: Supplementary file 2 [file Table_1.docx]

**Supplementary Table 1. Comparison of headache resolution rates and improvement rates based on the presence or absence of somnolence**

| **Improvement rate** | 30 min | 60 min | 120 min | 24 h |
| --- | --- | --- | --- | --- |
| somnolence + | (19/68) 27.9% | (29/59) 47.5% | (34/55) 61.8% | (37/70) 52.9% |
| somnolence − | (27/95) 28.4% | (44/90) 48.9% | (58/82) 70.7% | (72/92) 78.3% |
| p value | 0.947 | 0.864 | 0.276 | **<0.001** |

| **Resolution rate** | 30 min | 60 min | 120 min | 24 h |
| --- | --- | --- | --- | --- |
| somnolence + | (1/68) 1.5% | (9/59) 15.3% | (16/55) 29.1% | (27/70) 38.6% |
| somnolence − | ( 0/95) 0.0% | (15/90) 16.7% | (32/82) 39.0% | (47/92) 51.1% |
| p value | 0.417 | 0.819 | 0.232 | 0.113 |

Statistically significant values are shown in bold.
