# Supplementary material for: Real-world effectiveness and safety of lasmiditan in patients with migraine: an observational study
Source: Front Neurol. 2026 Sep 29;17:1968634. doi: 10.3389/fneur.2026.1968634 (PMC13657970; doi:10.3389/fneur.2026.1968634)
Supplement: Supplementary file 3 [file Table_2.docx]

**Supplementary Table 2. Comparison of headache resolution rates and improvement rates based on the presence or absence of weakness**

| **Improvement rate** | 30 min | 60 min | 120 min | 24 h |
| --- | --- | --- | --- | --- |
| weakness + | (12/53) 22.6% | (25/50) 50.0% | (29/47) 61.7% | (35/47) 74.5% |
| weakness − | (34/108) 31.5% | (54/98) 55.1% | (66/88) 75.0% | (88/106) 83.0% |
| p value | 0.243 | 0.514 | 0.107 | 0.219 |

| **Resolution rate** | 30 min | 60 min | 120 min | 24 h |
| --- | --- | --- | --- | --- |
| weakness + | (0/53) 0.0% | (7/50) 14.0% | (7/47) 14.9% | (13/47) 27.7% |
| weakness − | (1/108) 0.9% | (17/98) 17.3% | (41/88) 46.6% | (59/106) 55.7% |
| p value | >0.999 | 0.601 | **<0.001** | **0.001** |

Statistically significant values are shown in bold.
