# Supplementary material for: Real-world effectiveness and safety of lasmiditan in patients with migraine: an observational study
Source: Front Neurol. 2026 Sep 29;17:1968634. doi: 10.3389/fneur.2026.1968634 (PMC13657970; doi:10.3389/fneur.2026.1968634)
Supplement: Supplementary file 4 [file Table_3.docx]

**Supplementary Table 3. Changes in most bothersome symptoms after taking lasmiditan (n=72)**

|  | 30 min | 60 min | 120 min | 24 h |
| --- | --- | --- | --- | --- |
| **MBS resolution, n (%)** | 21 (29.2) | 41 (56.9) | 42 (58.3) | 51 (70.8) |
