# Supplementary material for: Real-world effectiveness and safety of lasmiditan in patients with migraine: an observational study
Source: Front Neurol. 2026 Sep 29;17:1968634. doi: 10.3389/fneur.2026.1968634 (PMC13657970; doi:10.3389/fneur.2026.1968634)
Supplement: Supplementary file 5 [file Table_4.docx]

**Supplementary Table 4. Patient characteristics**

|  | Included patients | Excluded patients | p |
| --- | --- | --- | --- |
| n (M/F) | 57 (13/44) | 18 (2/16) |  |
| Number of headaches | 180 | − |  |
| Age, years | 46.0±12.1 | 43.0±13.3 | 0.856 |
| Disease duration, years | 22.0±11.5 | 18.5±10.6 | 0.745 |
| Chronic migraine, n (%) | 20 (35.1) | 7 (38.9) | 0.770 |
| Migraine with aura, n (%) | 9 (15.8) | 3 (16.0) | 0.930 |
| MOH, n (%) | 20 (35.1) | 12 (16.0) | **0.018** |
| Accompanying symptoms, n(%) |  |  |  |
| Photophobia | 45 (78.9) | 14 (77.8) | 0.916 |
| Phonophobia | 44 (77.2) | 12 (66.7) | 0.371 |
| Osmophobia | 40 (70.2) | 9 (50.0) | 0.117 |
| Analgesics, n (%) |  |  |  |
| NSAIDs | 31 (54.4) | 13 (72.2) | 0.180 |
| Acetaminophen | 6 (10.5) | 2 (11.1) | 0.452 |
| Triptan | 42 (73.7) | 14 (77.8) | 0.728 |
| Migraine prophylaxis, n (%) |  |  |  |
| CGRP mAbs | 29 (50.9) | 10 (55.6) | 0.729 |
| Fremanezumab | 13 (22.8) | 6 (33.4) | 0.371 |
| Galcanezumab | 12 (21.1) | 3 (16.7) | 0.685 |
| Erenumab | 4 (7.0) | 1 (5.6) | 0.828 |
| Calcium channel blocker | 17 (29.8) | 4 (22.2) | 0.531 |
| Antiepileptics | 15 (26.3) | 3 (16.7) | 0.403 |
| Antidipressants | 11 (19.3) | 3 (16.7) | 0.803 |
| Lasmiditan dosage |  |  |  |
| 50mg | 39 (68.4) | 15 (83.3) | 0.219 |
| 100mg | 17 (29.8) | 2 (11.1) | 0.133 |
| 200mg | 1 (1.8) | 1 (5.6) | 0.425 |
| Comorbidities | 43 (75.4) | 15 (83.3) | 0.486 |
