# Supplementary material for: Real-world effectiveness and safety of lasmiditan in patients with migraine: an observational study
Source: Front Neurol. 2026 Sep 29;17:1968634. doi: 10.3389/fneur.2026.1968634 (PMC13657970; doi:10.3389/fneur.2026.1968634)
Supplement: Supplementary file 6 [file Table_5.docx]

**Supplementary Table 5.** Exploratory subgroup analyses of 2-hour and 24-hour headache outcomes according to chronic migraine and medication-overuse headache status

| Outcome | CM | EM | p value |
| --- | --- | --- | --- |
| Headache improvement at 2 h, n/N (%) | 36/53 (67.9%) | 68/95 (71.6%) | 0.641 |
| Headache improvement at 24 h, n/N (%) | 46/68 (67.6%) | 83/99 (83.8%) | **0.014** |
| Headache resolution at 2 h, n/N (%) | 14/53 (26.4%) | 36/95 (37.9%) | 0.157 |
| Headache resolution at 24 h, n/N (%) | 18/68 (26.5%) | 56/99 (56.6%) | **<0.001** |

| Outcome | MOH | Non-MOH | p value |
| --- | --- | --- | --- |
| Headache improvement at 2 h, n/N (%) | 31/46 (67.4%) | 67/92 (72.8%) | 0.507 |
| Headache improvement at 24 h, n/N (%) | 33/56 (58.9%) | 93/101 (92.1%) | **<0.001** |
| Headache resolution at 2 h, n/N (%) | 10/46 (21.7%) | 38/92 (41.3%) | **0.023** |
| Headache resolution at 24 h, n/N (%) | 10/56 (17.9%) | 64/101 (63.4%) | **<0.001** |
