# Supplementary material for: Real-world effectiveness and safety of lasmiditan in patients with migraine: an observational study
Source: Front Neurol. 2026 Sep 29;17:1968634. doi: 10.3389/fneur.2026.1968634 (PMC13657970; doi:10.3389/fneur.2026.1968634)
Supplement: Supplementary file 7 [file Table_6.docx]

**Supplementary Table 6.** Comparison of baseline clinical characteristics between patients with and without somnolence and between those with and without weakness

| Characteristics | Somnolence + | Somnolence - | p value | Weakness + | Weakness - | p value |
| --- | --- | --- | --- | --- | --- | --- |
| Number of headache | 106 | 74 | − | 84 | 96 | − |
| Age, years | 46.0±11.0 | 46. 0±13.6 | 0.986 | 43.5±11.4 | 47. 0±12.7 | 0.559 |
| Female, n (%) | 25 (75.8) | 19 (79.2) | 0.965 | 20 (76.9) | 24 (77.4) | 0.762 |
| Disease duration, years | 23.0±10.9 | 20.0±12.1 | 0.434 | 21.5±12.6 | 23.0±10.5 | 0.802 |
| Chronic migraine, n (%) | 12 (36.4) | 8 (33.4) | 0.813 | 9 (34.6) | 11 (35.5) | 0.945 |
| Migraine with aura, n (%) | 6 (18.2) | 3 (12.5) | 0.561 | 5 (19.2) | 4 (12.9) | 0.514 |
| MOH, n (%) | 4 (12.1) | 16 (66.7) | **<0.001** | 9 (34.6) | 11 (35.5) | 0.945 |
| Baseline NRS |  |  |  |  |  |  |
| Lasmiditan 50 mg, n (%) | 26 (78.8) | 13 (54.2) | **0.048** | 21 (80.8) | 18 (58.1) | 0.066 |
| Lasmiditan 100/200 mg, n (%) | 7 (21.2) | 10 (41.6) | 0.096 | 5 (19.2) | 12 (38.8) | 0.109 |
| CGRP mAb use, n (%) | 18 (54.6) | 11 (45.8) | 0.516 | 13 (50.0) | 15 (48.4) | 0.903 |
| Fremanezumab, n (%) | 7 (21.2) | 6 (25.0) | 0.784 | 6 (23.1) | 6 (19.4) | 0.731 |
| Galcanezumab, n (%) | 8 (24.2) | 4 (16.7) | 0.489 | 5 (19.2) | 7 (22.6) | 0.757 |
| Erenumab, n (%) | 3 (9.1) | 1 (4.2) | 0.472 | 2 (7.7) | 2 (6.5) | 0.855 |
| Antidepressants, n (%) | 6 (18.2) | 5 (20.8) | 0.802 | 7 (26.9) | 5 (16.1) | 0.319 |
| Calcium channel blockers, n (%) | 11 (33.3) | 6 (25.0) | 0.497 | 8 (30.8) | 4 (12.9) | 0.099 |
| Antiepileptics, n (%) | 10 (30.3) | 5 (20.8) | 0.423 | 6 (23.1) | 9 (29.0) | 0.611 |
